# ACPA: automated cluster plot analysis of genotype data

**DOI:** 10.1186/1753-6561-3-s7-s58

**Published:** 2009-12-15

**Authors:** Arne Schillert, Daniel F Schwarz, Maren Vens, Silke Szymczak, Inke R König, Andreas Ziegler

**Affiliations:** 1 Institut für Medizinische Biometrie und Statistik, Universität zu Lübeck, 23538 Lübeck, Germany

## Abstract

Genome-wide association studies have become standard in genetic epidemiology. Analyzing hundreds of thousands of markers simultaneously imposes some challenges for statisticians. One issue is the problem of multiplicity, which has been compared with the search for the needle in a haystack. To reduce the number of false-positive findings, a number of quality filters such as exclusion of single-nucleotide polymorphisms (SNPs) with a high missing fraction are employed. Another filter is exclusion of SNPs for which the calling algorithm had difficulties in assigning the genotypes. The only way to do this is the visual inspection of the cluster plots, also termed signal intensity plots, but this approach is often neglected. We developed an algorithm ACPA (automated cluster plot analysis), which performs this task automatically for autosomal SNPs. It is based on counting samples that lie too close to the cluster of a different genotype; SNPs are excluded when a certain threshold is exceeded. We evaluated ACPA using 1,000 randomly selected quality controlled SNPs from the Framingham Heart Study data that were provided for the Genetic Analysis Workshop 16. We compared the decision of ACPA with the decision made by two independent readers. We achieved a sensitivity of 88% (95% CI: 81%-93%) and a specificity of 86% (95% CI: 83%-89%). In a screening setting in which one aims at not losing any good SNP, we achieved 99% (95% CI: 98%-100%) specificity and still detected every second low-quality SNP.

## Background

Genome-wide association studies (GWAS) with 100,000-1,000,000 single-nucleotide polymorphisms (SNPs) are a promising novel approach for dissecting the genetic background of complex diseases and have become common in the last two years [1]. Because of the high degree of automation in the genotyping process, great care needs to be taken to generate high data quality [2]. Here, a number of quality criteria seem to be agreed upon, including a SNP-wise call fraction or the conformation of genotype frequencies with Hardy-Weinberg equilibrium [3,4].

Another important criterion is the quality of the results from the calling algorithm, which transforms the fluorescence signal intensities into one of the three possible genotypes. For evaluation, the visual inspection of signal intensities through cluster plots, also termed signal intensity plots, has been recommended [4,5], and the validity of the genotype assignment may be assessed. Our experience with previously performed GWAS of coronary artery disease [6,7] has shown that the gold standard for this evaluation as of yet is the visual inspection of the cluster plots by at least two independent and experienced readers. This is very time-consuming and depends on the training and availability of experienced readers. Because of this, only a selection of interesting SNPs in a GWAS are usually evaluated. Previous analyses have shown that erroneous genotype scoring can lead to false-positive or false-negative associations, so that the number of low-quality SNPs may be overestimated through this selection [2].

There are two principal approaches to tackle this problem. The first focuses on improving the calling algorithm itself [8]. The second, which is followed here, aims at automating the evaluation of the cluster plots. We have developed an algorithm called automated cluster plot analysis (ACPA) that fulfills four requirements: 1) reduction of work load: out of a vast number of genotyped SNPs, ACPA classifies only a small proportion to have questionable quality and thus need to be inspected visually; 2) high negative predictive value: of the SNPs classified to be of high quality by ACPA, only a small portion is erroneously classified; 3) reasonable speed: on a simple personal computer, 1,000 SNPs are analyzed in approximately 10 minutes, and the processes may also be split between different machines; and 4) user-friendly environment: ACPA was implemented in R using the GenABEL [9] library.

## Methods

### Data

Signal intensities and genotypes of 6,752 participants in the Framingham Heart Study were provided as Problem 2 for the Genetic Analysis Workshop 16 (GAW16).

Genotyping was performed using the Affymetrix GeneChip Human Mapping 500 k

Array Set. Details of the study and the genome-wide SNP scan can be found in Cupples et al. [10].

### Algorithm

The ACPA algorithm, which analyses one SNP at a time, is described below for the case of exactly three different clusters, i.e., three genotype groups.

1. Select all individuals with assigned genotypes, i.e., delete all missing genotypes.

2. For clusters *k *= 1 to 3:

a) Perform a principal-component analysis, using only data of cluster *k*.

b) Transform the data of all clusters according to estimated first two principal components of step a).

c) Calculate the Mahalanobis distance from the center of cluster k to all samples within cluster k.

d) Define the cluster boundary *b *as *b *= *q*_3 _+ *f. *IQR, where *q*_3 _is the upper quartile and IQR is the interquartile range of the Mahalanobis distances computed in step c). Default: *f *= 3.

e) Calculate the Mahalanobis distance from the center of cluster *k *to the samples not included in *k*.

f) Count the number *c*_*k*_, of samples not included in *k *falling in the boundary.

3. Sum the number of samples falling in the boundary over all three clusters, i.e., *c *= ∑ *c*_*k*_

4. A SNP is called to have unreliable genotype assignments if *c *exceeds a threshold t. Default: *t *= 25.

If the SNP has a low minor allele frequency leading to two clusters, the algorithm is adapted appropriately. The validation status *c *of each cluster-plot is logged in a file. The algorithm has been implemented in R (version 2.7.1) and uses the library GenABEL (version 1.4-1) for storing the genotype data. The cluster plot can be generated together with the cluster boundaries in portable document format (pdf).

**Figure 1:**
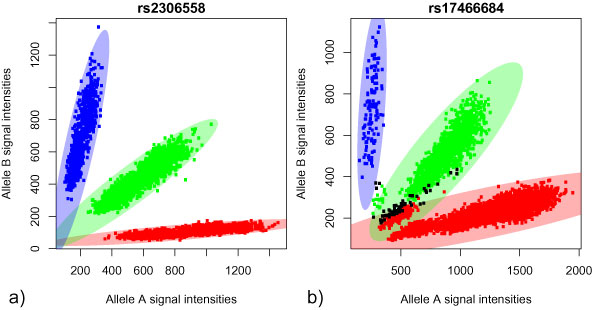
**Examples of cluster plots**. Cluster plots for two SNPs. One spot corresponds to one sample. Samples with genotypes AA and BB are red and blue, respectively. Heterozygous samples are shown in green; samples with missing genotypes are black. The ellipses represent the cluster boundaries as computed by ACPA. a, A SNP with no samples in overlapping ellipses; b, red samples lie in the green ellipse. At the bottom of the green ellipse, samples have been erroneously classified as red samples.

Two example plots are displayed in Figure 1. Figures 1, a and 1, b, show SNPs with clearly separated clusters and with bad separation of clusters, respectively.

By using different values for the factor *f *(see above, step 2d), the size of the ellipses (Figure 1) can be changed. Specifically, lowering *f *will lead to smaller ellipses, resulting in fewer samples within the boundary. Using *f *= 1.5 follows a commonly used definition of outliers when considering quartiles and the interquartile range. Around each cluster an ellipse will be constructed and the number of potentially misclassified samples is counted. The sum over all three clusters is then compared with a cut-off value *t*. This parameter depends on the sample size. For this particular data set we chose *t *= 25, which maximizes the accuracy of ACPA.

### Evaluation of the algorithm

The performance of the ACPA algorithm was evaluated by comparing ACPA with the decisions made by two experienced readers. First, we performed standard quality control (sQC) and omitted SNPs with deviations from Hardy-Weinberg-equilibrium (*p *< 10^-4 ^for exact lack-of-fit test), missing fraction >0.02, and minor allele frequency <0.01. We randomly selected 1,000 SNPs of the remaining SNPs. Both readers judged independently whether a SNP should be excluded from or kept for further analyses. They also gave a level of uncertainty (certain/uncertain) of their decisions. Because we cannot expect ACPA to outperform the reading of experienced readers, we only considered SNPs for which both readers came to the same decision and expressed certainty. A good SNP is a SNP when both readers recommend keeping that SNP for further analysis. If both readers favor the exclusion of a SNP, we call this a bad SNP.

We report sensitivity, specificity, positive predictive values (PPV) and negative predictive values (NPV) of ACPA as well as 95% confidence intervals (95% CI) using Wilson's score method [11]. Here, sensitivity denotes the proportion of correctly identified bad clustering SNPs, and specificity is the proportion of correctly identified good clustering SNPs. We evaluated the performance of ACPA for two different ellipse sizes (*f *= 1.5 and *f *= 3).

## Results

Out of the 486,605 BRLMM-called SNPs that were provided for GAW16, 343,427 SNPs successfully passed the sQC. For 695 of the 1,000 randomly selected SNPs, the two independent readers came to identical decisions, and both readers expressed certainty about their decision. Five hundred and eighty-eight (84.6%) were judged as correctly called SNPs, and the remaining 107 SNPs were classified as SNPs where genotype assignment was unreliable. Point and interval estimates for sensitivity, specificity, PPV, and NPV are shown in Table 1. For *f *= 1.5 we achieved a specificity of 99%, i.e., almost all good SNPs were recognized by ACPA.

**Table 1:** Quality of the automatic analysis with ACPA

	Point estimate [CI]			
	
Cluster boundary *f*	Sensitivity	Specificity	PPV	NPV
1.5	0.51 [0.41, 0.60]	0.99 [0.98,1.00]	0.93 [0.83,0.97]	0.92 [0.90,0.94]
3	0.88 [0.81,0.93]	0.86 [0.83,0.89]	0.53 [0.45,0.60]	0.98 [0.96,0.99]

Increasing the cluster boundaries resulted in a loss of specificity (86%) but increased the sensitivity of ACPA to detect badly clustered SNPs. The PPV declined from 93% to 53%.

## Discussion

For the randomly selected sample of 1,000 SNPs, both readers recommended the exclusion of 107 SNPs. Although data quality may vary, there will always be thousands of SNPs that should be excluded. This shows the necessity of analyzing cluster plots. The large amount of SNPs requires an automatic approach, and this is provided by ACPA. Because it would not be reasonable to exclude well clustered SNPs at the screening stage, the method must be highly specific. This can be achieved by choosing a small *f*, resulting in narrow ellipses and hence only excluding very badly assigned clusters. For example, the specificity is 99% for *f *= 1.5. Of course, this comes at the cost of keeping many doubtful SNPs in the data (sensitivity = 51%). In a situation where it would be costly to include SNPs with a bad cluster plot, one could increase the value for *f *to exclude doubtful SNPs.

## Conclusion

The necessity of visually inspecting cluster assignments from the genotype calling was demonstrated, and an intuitive approach was proposed for performing this task automatically. As a by-product, our novel software ACPA provides a convenient way to generate cluster-plots for any subset of SNPs.

## List of abbreviations used

ACPA: Automated cluster plot analysis; GAW16: Genetic Analysis Workshop 16; GWAS: Genome-wide association study; NPV: Negative predictive values; PPV: Positive predictive values; SNP: Single-nucleotide polymorphism; sQC: Standard quality control.

## Competing interests

The authors declare that they have no competing interests.

## Authors' contributions

AS carried out the analysis, participated in writing ACPA, and drafted the manuscript. DFS participated in writing ACPA. MV and SS rated the SNPs. IRK participated in the design and coordination of the study. AZ conceived of the study and finalized the manuscript. All authors read and approved the final manuscript.
